# Continuous Intravenous Insulin Infusion in Patients with Diabetes Mellitus After Coronary Artery Bypass Grafting: Impact on Glycemic Control Parameters and Postoperative Complications

**DOI:** 10.3390/jcm14093230

**Published:** 2025-05-06

**Authors:** Alexey N. Sumin, Natalia A. Bezdenezhnykh, Dmitry L. Shukevich, Andrey V. Bezdenezhnykh, Olga L. Barbarash

**Affiliations:** 1 Federal State Budgetary Scientific Institution, Research Institute for Complex Issues of Cardiovascular Diseases, Academician L.S. Barbarash Boulevard, 6, Kemerovo 650002, Russia; 746701@mail.ru (D.L.S.); olb61@mail.ru (O.L.B.); 2 Limited Liability Company “Family Health and Reproduction Center Krasnaya Gorka”, Suvorova st., 3A, Kemerovo 650044, Russia; andrew22014@mail.ru; 3 Department of Cardiology and Cardiovascular Surgery, Federal State Budgetary Educational Institution of Higher Education “Kemerovo State Medical University”, Voroshilova st., 22A, Kemerovo 650056, Russia

**Keywords:** type 2 diabetes mellitus, perioperative glycemic management, short-acting insulin, postoperative complications

## Abstract

**Objectives:** This study compared the efficacy of continuous insulin infusion therapy (CIT) versus standard bolus insulin therapy in maintaining optimal perioperative glycemic control in patients with type 2 diabetes mellitus (T2DM) undergoing coronary artery bypass grafting (CABG), focusing on postoperative outcomes. **Methods:** In this single-center, open comparative study, 214 T2DM patients were selected from 1372 CABG cases (2016–2018) and divided into CIT (*n* = 28) and bolus therapy (*n* = 186) groups. Both groups were matched for sex, age, smoking status, body mass index, functional class of angina or heart failure, surgical characteristics and preoperative HbA1c. The target glucose range was 7.8–10 mmol/L (140–180 mg/dL), consistent with current guidelines. Glycemic control was assessed through frequent postoperative measurements, with particular attention to glucose variability and hypoglycemic events. **Results:** The CIT group demonstrated superior glycemic control, with significantly lower median glucose levels at 7, 8, 10, 12, and 13 h post-CABG (*p* < 0.05). Glycemic variability was reduced by 32% in the CIT group (*p* = 0.012), and the incidence of hypoglycemia (<3.9 mmol/L) was 3.6% versus 8.1% in the bolus group. While overall complication rates were similar, the CIT group had 0 cases of stroke, myocardial infarction, or wound infections versus 2.7%, 3.2%, and 5.9%, respectively, in the bolus group. Logistic regression confirmed that each 1 mmol/L increase in first-day glucose levels independently predicted both significant (OR 1.20, 95% CI 1.06–1.36) and serious complications (OR 1.16, 95% CI 1.03–1.30). **Conclusions:** CIT provided more stable postoperative glycemic control with reduced variability and hypoglycemia risk in T2DM patients after CABG. Although underpowered to detect differences in rare complications, our findings suggest CIT may improve outcomes. These results warrant validation in larger randomized trials.

## 1. Introduction

Coronary artery bypass grafting (CABG) is an effective method of revascularization in patients with diabetes mellitus and coronary artery disease (CAD) [1]. Type 2 diabetes mellitus (T2T2DM) patients account for 25 to 40% of those referred for cardiac surgery, and their proportion is constantly growing [1,2]. Moreover, diabetes is both a marker of high-risk, resource-intensive, and expensive treatment after CABG, and an independent risk factor for decreased long-term survival [3,4]. Moreover, uncontrolled blood glucose levels in patients with diabetes are associated with adverse perioperative outcomes [5]. Perioperative hyperglycemia per se, even in patients without diabetes, is associated with adverse outcomes after cardiac surgery [6,7,8]. Accordingly, perioperative glycemic control is critical to maintain favorable clinical outcomes [9].

Observational studies have found a U-shaped association between mean blood glucose levels and death, with the lowest mortality rates observed in the range of 125–160 mg/dL [5,7]. Accordingly, data indicate an increased risk of hypoglycemic events with aggressive glycemic control and suggest that moderate control can achieve clinically meaningful improvements [5]. Currently, the target glycemic values for intensive care unit patients defined in the guidelines are 7.8–10.0 mmol/L (140–180 mg/dL) without hypoglycemia [10,11,12]. Experts believe that the best way to achieve glycemic control goals is continuous intravenous insulin infusion [10,11]. However, despite evidence of the advantage of this method of glycemic control over standard therapy with bolus insulin [13,14], it still not only prevails in routine clinical practice, but is also widely used even in studies examining the effect of perioperative control on perioperative complications [5,7,15]. Since the method of continuous intravenous insulin infusion in coronary artery bypass grafting has not yet become routine in Russian clinical practice, studies on the advantages of this method of glycemic control in CABG are in demand.

There is also evidence that it is necessary to study not only the blood glucose level upon admission to the intensive care unit in patients with diabetes mellitus who have undergone coronary artery bypass grafting, but also additional indices associated with glucose levels. Among such indices, the mean blood glucose level, mean absolute glucose level, mean amplitude of glycemic fluctuations, glycemic lability index and the largest amplitude of glycemic fluctuations (LAGE) are considered [16]. Accordingly, the aim of this study was to compare perioperative glycemic control strategies in patients with type 2 diabetes mellitus on the first day after coronary artery bypass grafting (continuous intravenous insulin infusion therapy and standard therapy with bolus insulin administration) in terms of their impact on glycemic indices and hospital complications.

## 2. Methods

### 2.1. Study Population

**Figure 1:**
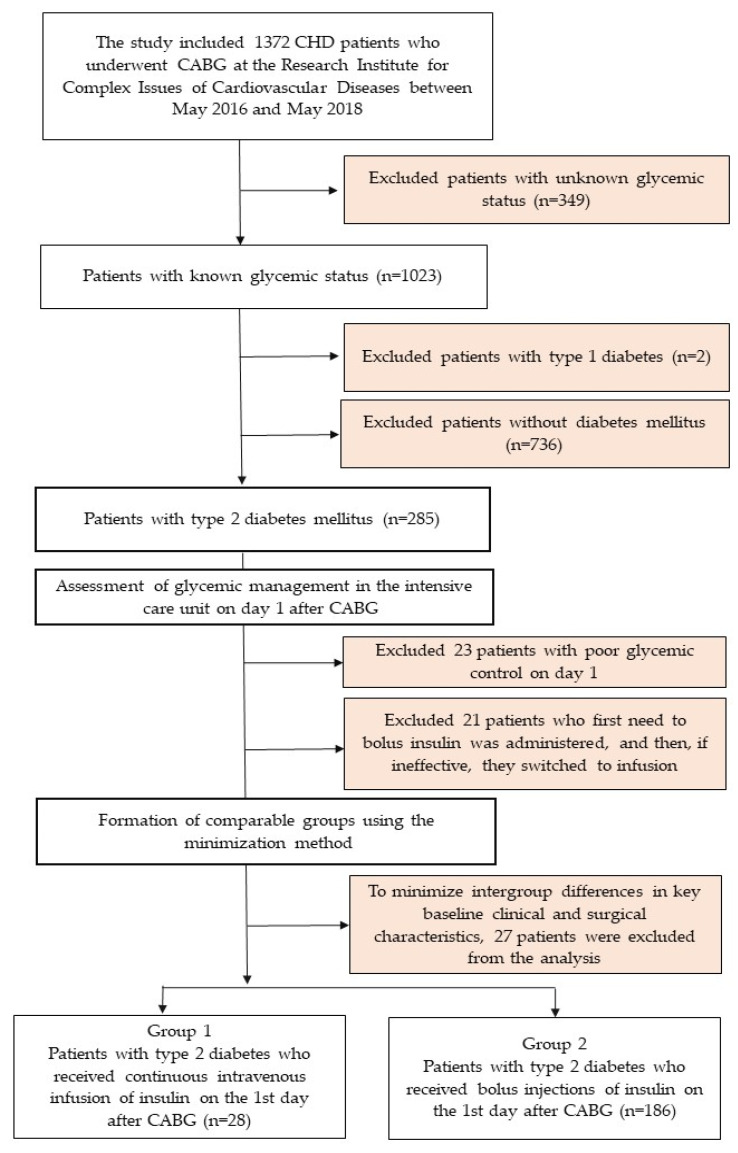
Flow chart of patient inclusion in the study. Notes: CHD—coronary heart disease; CABG—coronary artery bypass grafting.

A single-center, open comparative study was conducted. The study included all patients who underwent planned coronary artery bypass grafting (CABG) from 1 May 2016 to 1 May 2018 in the surgical clinic of the Research Institute for Complex Issues of Cardiovascular Diseases (Kemerovo, Russian Federation) and were included in the CABG registry. In total, 1372 patients were operated on during this time, of which 1023 patients with known glycemic status were selected. Then, 2 patients with a diagnosis of type 1 diabetes and 736 patients without type 2 diabetes were excluded, thus forming a sample of 285 patients with an established diagnosis of type 2 diabetes (Figure 1). Demographic and clinical characteristics of the patients and the results of inpatient treatment were obtained by analyzing medical records. The study was conducted in accordance with the principles of the Declaration of Helsinki. All patients signed informed consent to participate in the study. The study protocol was approved by the meeting of the Local Ethics Committee of the Research Institute for Complex Issues of Cardiovascular Diseases with an extract from protocol No. 7 dated 27 April 2017.

On the first postoperative day following CABG, all patients with glucose levels above target values received short-acting insulin therapy upon ICU admission (administered intravenously, subcutaneously, or as continuous intravenous infusion). Perioperative glycemic targets were maintained at 7.8–10 mmol/L (140–180 mg/dL) in accordance with current clinical guidelines [17]. Patients were randomly allocated to either (1) the continuous insulin infusion therapy (CIT) group, or (2) standard glycemic management group (receiving intravenous bolus insulin plus subcutaneous insulin in a 1:4 ratio of CIT to bolus therapy). The treating intensivist determined the insulin regimen randomly following this allocation ratio. Subsequent group composition analysis was performed according to the parameters described below. The CIT protocol strictly followed the algorithm specified in reference [17]. Following transfer to the cardiac surgery ward, all patients transitioned to subcutaneous insulin therapy combining short-acting and basal insulin formulations, with regimens individualized by endocrinologists based on daily glycemic control. Glycemic monitoring during the first day after surgery in the intensive care unit was per-formed in most cases once every 2 h, and once every 4 h when glycemia stabilized in the target ranges (7.8–10.0 mmol/L or 140–180 mg/dL). In the CIT group, venous blood glucose monitoring was performed using the hexokinase method once an hour, and once every two hours when glycemia stabilized in the target ranges (7.8–10.0 mmol/L or 140–180 mg/dL). Thus, patients had blood glucose measurement results from 6 to 24 times during the first postoperative days.

During group formation, we excluded patients who initially received short-acting insulin bolus therapy in the ICU but required conversion to intravenous insulin infusion due to treatment failure (defined as two consecutive glucose measurements >13.9 mmol/L; n = 23). We additionally excluded patients who demonstrated persistent hyperglycemia yet either received no insulin or received demonstrably inadequate doses, thereby meeting criteria for unsatisfactory glycemic control (n = 21).

The comparison groups were established using minimization methodology, ensuring balanced distribution of participants between treatment arms while maintaining comparability across key clinical parameters: sex, age, surgical risk (EuroSCORE II), CABG duration, combined surgical procedures, renal function, and baseline functional classification of both heart failure and angina pectoris. To further optimize baseline characteristic matching, 27 additional patients were excluded during this allocation phase.

The final analysis included 214 patients with type 2 diabetes mellitus, 28 of whom received non-invasive insulin therapy and 186 received bolus insulin therapy for glycemic control.

The individual average glucose level (arithmetic mean) and its variability (calculated using the standard deviation) were assessed within the first 24 h after coronary artery bypass grafting. The time was measured from the moment of transfer from the operating room to the intensive care unit. The largest amplitude of glycemic excursions (LAGE), an indicator of blood glucose fluctuations, was determined as the difference between the maximum and minimum blood glucose levels on the first postoperative day. The proportion of patients with hypoglycemia (any episode of glycemia below 3.9 mmol/L (70 mg/dL) and the proportion of patients with an average glucose level on the first day were assessed, as follows: (1) in the range of 7.8–10.0 mmol/L (140–180 mg/dL), (2) above 10 mmol/L (180 mg/dL) and (3) above 13.9 mmol/L (250 mg/dL).

### 2.2. Clinical Outcomes

This study evaluated the frequency and predictors of in-hospital postoperative complications following CABG. Serious complications were defined as in-hospital mortality, myocardial infarction, heart failure requiring inotropic support, percutaneous coronary intervention for perioperative acute coronary syndrome, cardiac arrhythmias, stroke, mesenteric thrombosis (1 case), emergency lower extremity vascular surgery for acute limb ischemia (1 case), multiple organ failure, extracorporeal hemofiltration, gastrointestinal bleeding, and surgical wound complications including wound dehiscence, edge necrosis, purulent infections requiring reoperation with secondary closure, or resternotomy for mediastinitis/hemorrhage. Significant complications comprised all serious complications listed above plus transient ischemic attacks, pneumonia, respiratory failure, and therapeutic pleural drainage for hydrothorax or pneumothorax.

The primary outcome was the occurrence of any serious postoperative complication. The secondary outcome was the occurrence of any significant in-hospital complication. The tertiary outcome was failure to achieve target glycemic values on the first day after CABG. The time frame for assessing treatment outcomes in terms of target glycemic values was limited to the first 24 h after CABG, during which time CIT was used. For serious or significant complications, outcomes were assessed until hospital discharge, but no later than 30 days post-surgery.

### 2.3. Statistical Analysis

Statistical processing was performed using the standard software packages “STATISTICA 8.0” (Dell Software, Inc., Round Rock, TX, USA) and SPSS 17.0 (IBM, Armonk, NY, USA). The normality of distribution of quantitative data was checked using the Shapiro–Wilk criterion. For pairwise comparison of groups, the Mann–Whitney criterion and χ2 (chi-square) were used. For a small number of observations, Fisher’s exact test with Yates’ correction was used. Logistic regression analysis was used to identify predictors of adverse outcomes. The level of critical significance (*p*) in the regression analysis was taken to be 0.05.

## 3. Results

General characteristics of patients in the CIT and standard therapy groups are presented in Table 1. In both groups, the majority were men; the groups did not differ in median age, frequency of newly diagnosed diabetes mellitus, smoking status, body mass index, functional class of angina and heart failure, severity of coronary artery disease according to CAG or frequency of brachiocephalic artery stenosis (Table 1). Most operations were performed under artificial circulation. The main characteristics of CABG are presented in Table 1, and they were comparable in both groups.

The two groups demonstrated comparable baseline characteristics regarding preoperative therapy, including antihyperglycemic treatment regimens. Both groups showed similar frequencies of oral antidiabetic medication use (metformin, sulfonylureas, DPP-4 inhibitors, SGLT-2 inhibitors, and GLP-1 agonists) and comparable patterns of insulin administration both before and during hospitalization, with no statistically significant differences (*p* > 0.05; Table 1). Consistent with the treatment protocol, the total 24 h insulin requirement was significantly higher in the intravenous infusion group than in the bolus therapy group (*p* < 0.001).

**Figure 2:**
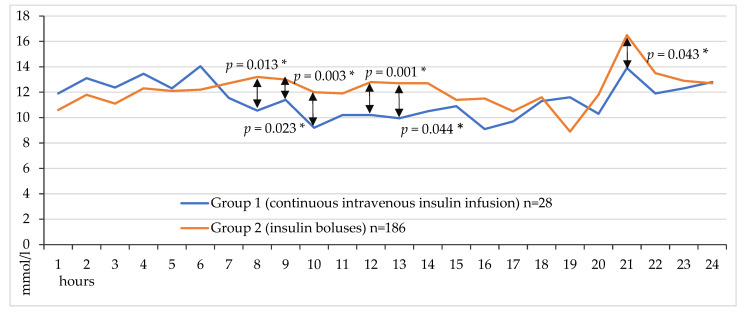
Hourly venous blood glucose levels on the first day after CABG.

The line diagram demonstrates glycemic fluctuations over 24 h after coronary artery bypass grafting in 2 groups (Figure 2). Median glucose was significantly lower without hypoglycemia in the CIT group (*p* = 0.013, *p* = 0.023, *p* = 0.003, *p* = 0.001, *p* = 0.044 and *p* = 0.043 for 8, 9, 10, 12, 13 and 21 h after completion of CABG, respectively, as shown in Figure 2.

**Figure 3:**
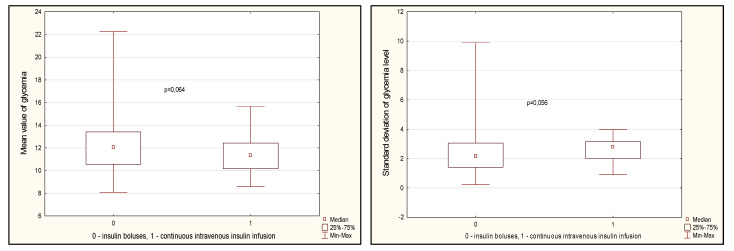

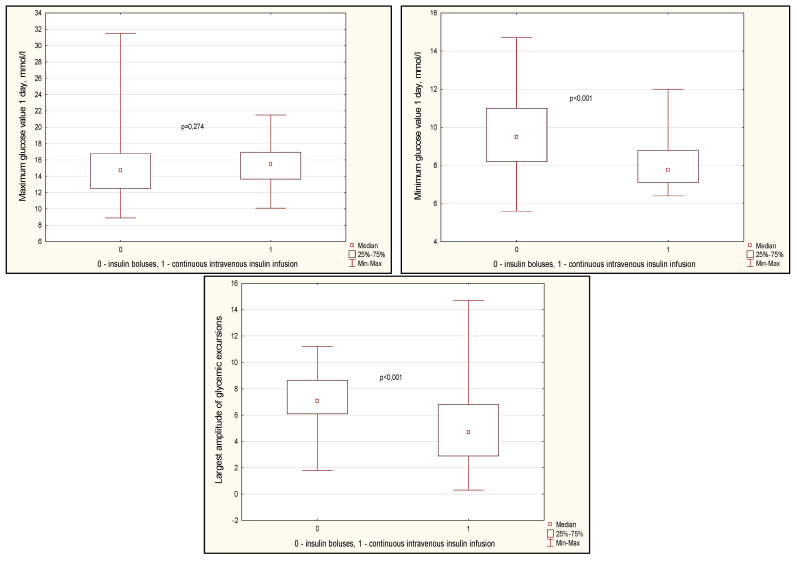
Glycemia variability indicators in groups on day 1 after CABG. Note: CABG—coronary artery bypass grafting, Me—median, LQ—lower quartile; UQ—upper quartile.

Glycemic variability indicators are presented in Figure 3. There was a tendency towards a lower value of average glycemia on the first day (Figure 3, *p* = 0.064). At the same time, the standard deviation of glucose fluctuations was slightly higher in the CIT group (*p* = 0.056). The maximum values of glucose levels on the first day did not differ between the groups, but the minimum values were lower in the CIT group (*p* < 0.001). At the same time, the range between the maximum and minimum glucose values was also lower in the CIT group (Figure 3, *p* < 0.001).

**Figure 4:**
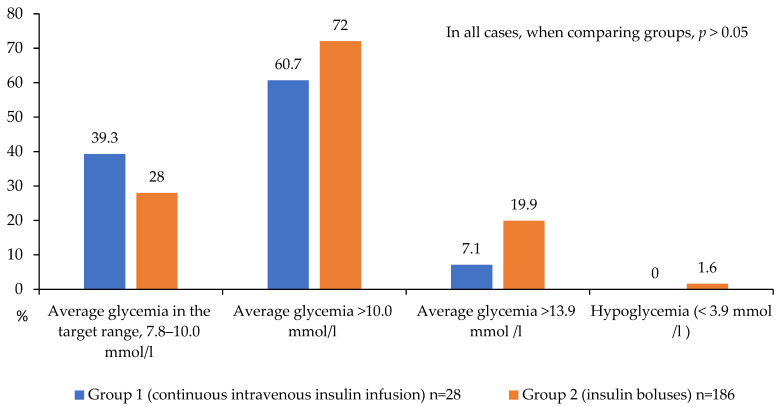
Achievement and non-achievement of target glycemia values on the 1st day after CABG.

Group 1 demonstrated a trend toward a lower proportion of patients with severe hyper-glycemia (>13.9 mmol/L) compared to the bolus insulin group (7.1% vs. 19.9%, respectively; *p* = 0.078, Figure 4). Target glucose levels during the first 24 h were achieved in 39.3% of patients receiving continuous intravenous insulin infusion (CIII) versus 28.0% in the control group, suggesting a trend toward better glycemic control with CIII. However, a substantial proportion of patients in both groups failed to reach target values, with mean glucose levels exceeding 10 mmol/L (60.7% and 72% in Groups 1 and 2, respectively; Figure 4). Hypoglycemic events (<3.9 mmol/L) were rare and occurred exclusively in Group 2 (1.6% of cases).

**Figure 5:**
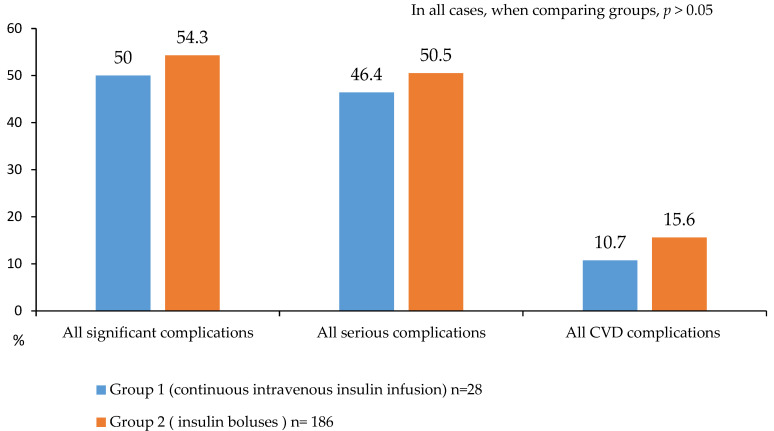
Serious and significant complications after CABG in 2 groups.

**Figure 6:**
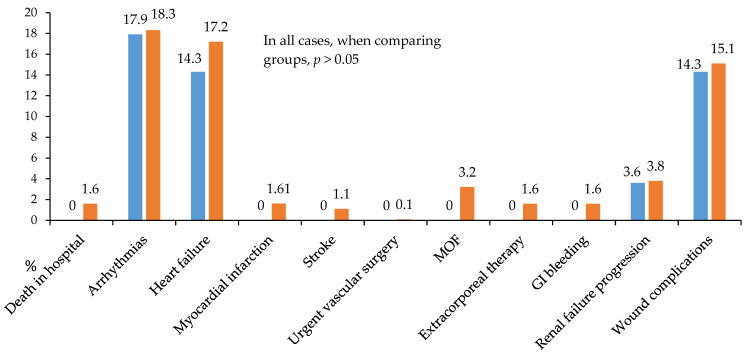
All postoperative hospital complications in 2 groups. Notes: GI—gastrointestinal; MOF—multiple organ failure.

**Figure 7:**
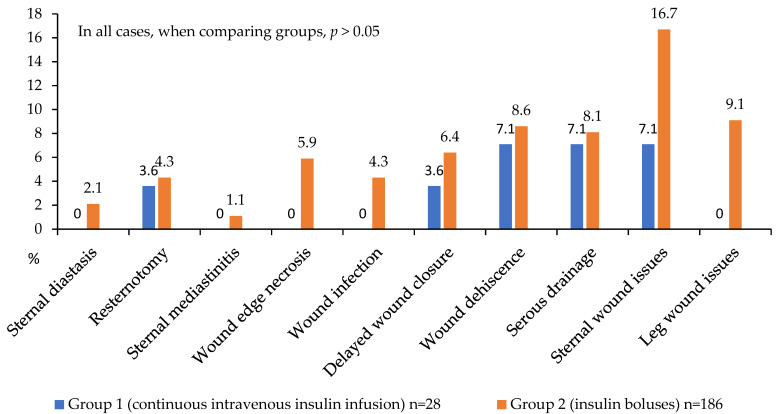
Postoperative wound complications in 2 groups.

A detailed analysis of in-hospital postoperative complications is presented in Figure 5, Figure 6 and Figure 7. While no statistically significant differences in complication rates were observed between the groups, both groups of diabetic patients demonstrated a high incidence of significant and serious complications (approximately 50% of cases; Figure 5).

Notably, the continuous insulin infusion therapy (CIIT) group showed no cases of in-hospital mortality, postoperative stroke, myocardial infarction, multiple organ failure, gastrointestinal bleeding, or need for extracorporeal hemocorrection (Figure 6).

Regarding wound complications, the CIT group had no instances of mediastinitis, sternal dehiscence, wound infection, or wound edge necrosis (Figure 7). In terms of complication localization, this group also exhibited no lower leg wound complications following vein harvesting, while sternal wound complications occurred in 16.7% versus 7.1% in the comparison group (*p* > 0.05). These findings suggest a trend toward more favorable postoperative outcomes in patients receiving CIT during the first 24 h. However, these intergroup differences did not reach statistical significance, likely due to the limited number of analyzed events (Figure 7).

We used binary logistic regression (direct LR method) to identify factors associated with the development of hospital complications. The model included the studied glycemic indicators obtained during glycemic control monitoring during the day, as well as the method of insulin therapy on the 1st day after surgery. Only one of the indicators studied in the models was associated with the development of both significant complications and serious complications for average glycemia on the 1st day (B = 0.185, *p* = 0.004 and B = 0.144, *p* = 0.019, respectively) (Table 2). For these models, the statistical significance was χ2(1) = 9.207, *p* = 0.002 and χ2(1) = 5.762, *p* = 0.016, respectively.

## 4. Discussion

In this study, we showed that continuous insulin infusion compared to bolus administration in patients with diabetes after CABG contributes to better postoperative glycemic control, in particular by reducing the range between the maximum and minimum glucose values and reducing the minimum glucose value during the day. We were unable to identify a significant reduction in the number of hospital complications in the continuous insulin infusion group, but the average glucose level during the first day was associated with the development of both significant and serious complications.

Recently published studies have shown the importance of an indicator such as the variability of glucose levels in the postoperative period as a factor determining the development of hospital complications [15]. Thus, after on-pump CABG, patients with diabetes with LAGE ≥ 4.4 mmol/L had significantly higher rates of the combined endpoint and major vascular complications [15]. It has been previously shown that this indicator (LAGE), along with the standard deviation of glucose fluctuations, can be used to effectively characterize glycemic variability associated with beta-cell function in patients with type 2 diabetes [18]. Our study convincingly demonstrated that the continuous insulin infusion method can effectively reduce the LAGE indicator. It was also shown that higher levels of average daily glucose and glycemic lability index in patients with diabetes after CABG were associated with a higher risk of developing postoperative delirium [16]. These data are consistent with our data on the independent association of the average glucose level during the first day after CABG with the development of hospital complications.

The method of continuous insulin infusion seems to be the optimal way to control glycemia in the postoperative period [13,14]. Thus, in the study by Golukhova EZ et al. [14], it was shown that continuous insulin infusion improves glycemic control and can potentially reduce the risk of the most common complication after CABG-post-pericardiotomy syndrome. In another study, CIT reduced the variability of glucose concentrations and the occurrence of deep sternal wound infection after CABG in a certain group of patients with diabetes with bimammary bypass grafting [13]. However, it was not always possible to prove the benefits of continuous insulin infusion. For example, it was not possible to identify the effect of this treatment method on the incidence of surgical site infections among diabetic patients with CABG [19]. These data show that improved glycemic control after surgery is not always accompanied by a decrease in the number of complications (as in our case). Conflicting data may be due to differences in the timing of interventions, in the working definitions adopted for the treatment groups, or in the acceptable level of hyperglycemia allowed in conventional groups [19].

We see the clinical significance of this study in confirming the effectiveness of glycemic control in the postoperative period in patients with diabetes in Russian conditions, i.e., primarily a significant decrease in glucose variability. These data may well help to overcome therapeutic inertia in practicing physicians. It is also worth mentioning the possibility of supplementing insulin therapy with Liraglutide, a glucagon-like peptide-1 receptor agonist. This combination can reduce both perioperative blood glucose levels and glycemic variability in the postoperative period in patients with diabetes mellitus undergoing cardiac surgery [20,21]. This has been shown both with bolus insulin administration [20] and with its continuous intravenous infusion [21]. Another possible way to improve glycemic control in patients with diabetes mellitus is presented in the publications of Shi et al. In patients with diabetes and acute myocardial infarction, the intervention of a clinical pharmacist contributed to improved results, in particular, a decrease in blood glucose fluctuations and the potential risk of hypoglycemia [22,23]. We believe that such an approach is possible in further management of patients after the first day after CABG surgery. In addition, studies are currently ongoing to study the mechanisms of development of glucose variability in the perioperative period, although one such study failed to show an association of postoperative glycemic variability with endocan, a marker of endothelial dysfunction [24]. However, a study is planned to examine the association of variability with perioperative changes in ANS activity in diabetic and non-diabetic patients undergoing cardiac surgery and the association between postoperative GV and blood and urine inflammation levels, as well as endothelial dysfunction biomarkers [20].

When considering the results of the present study, its limitations should be taken into account. First, this study was conducted in a single center and its results will be difficult to generalize to other clinics. Second, the choice of insulin therapy was not randomized and this choice was left to the discretion of the attending physician, which could potentially lead to a bias effect. However, a comparison of baseline and perioperative characteristics of patients in both groups did not reveal significant differences, so the lack of randomization does not seem to affect the significance of the results. Third, a small number of patients were included in the continuous insulin infusion group compared to the bolus insulin group. This is explained primarily by the inertia of thinking of doctors who preferred a more familiar type of insulin therapy. Perhaps this somewhat affected the statistical significance of the differences we obtained for some glycemic indicators and in the frequency of hospital complications. However, even the results obtained, in particular the reduction in the variability of glucose levels against the background of constant insulin infusion, are an additional argument for practicing doctors in favor of this method of therapy.

## 5. Conclusions

In this study, we have shown that continuous insulin infusion, compared with bolus administration in patients with diabetes after CABG, promotes better control of postoperative glycemia. Although this study failed to reveal a significant reduction in the number of hospital complications in the continuous insulin infusion group, the indicator characterizing glycemic control (average glucose level during the first day) was associated with the development of hospital complications. These results deserve continuation in further studies, both multicenter and randomized.

## Figures and Tables

**Table 1:** Baseline characteristics of the study groups (n = 214).

Indicator	Group 1 (Continuous Intravenous Insulin Infusion) n = 28	Group 2 (Insulin Boluses) n = 186	*p*
Male, n (%)	16 (57.1)	114 (61.3)	0.448
Female, n (%)	12 (42.9)	73 (38.7)	0.448
Age, years, Me [LQ; UQ]	64.8 [60.3; 67.0]	64.2 [59.9; 69.0]	0.941
Body mass index, Me [LQ; UQ]	27.8 [24.7; 31.1]	28.7 [25.9; 31.7]	0.577
Newly diagnosed type 2 diabetes, n (%)	5 (17.8)	54 (29.0)	0.217
Unstable angina, n (%)	0 (0)	2 (1.1)	0.994
FC 2 angina, n (%)	18 (64.2)	91 (48.9)	0.345
FC 3 angina, n (%)	6 (21.4)	36 (19.4)	0.786
Chronic heart failure FC 3 NYHA, n (%)	2 (7.1)	15 (8.2)	0.313
Chronic heart failure FC 2 NYHA, n (%)	26 (92.9)	166 (89.2)	0.359
History of stroke, n (%)	2 (7.14)	10 (5.1)	0.653
History of PCI, n (%)	3 (10.7)	19 (10.2)	0.922
History of CABG, n (%)	0 (0)	1 (0.5)	0.690
Smoking status, n (%)			
Smoker at the time of CABG, n (%)	0 (0)	6 (3.2)	0.717
Never smoked, n (%)	22 (84.2)	148 (79.5)	0.454
Quit, n (%)	5 (21.4)	32 (17.2)	0.610
Three-vessel disease by coronary angiography, n (%)	16 (57.1)	94 (50.5)	0.514
Left main coronary artery disease, n (%)	4 (14.3)	25 (13.4)	0.861
Stenosis of the brachiocephalic arteries, n (%)	7 (25.0)	46 (25.3)	0.838
Left ventricular ejection fraction, Me [LQ; UQ]	64.0 [50.0; 67.0]	62.0 [52.0; 66.0]	0.304
CKD-EPI glomerular filtration rate, Me [LQ; UQ]	75.8 [71.6; 92.5]	91.9 [76.7; 99.0]	0.087
HbA1c before CABG, Me [LQ; UQ]	7.4 [6.2; 8.8]	6.9 [6.0; 7.7]	0.224
Fasting blood glucose before CABG, Me [LQ; UQ]	6.7 [5.3; 7.3]	7.3 [6.1; 8.7]	0.331
EuroSCORE 2 (%), Me [LQ; UQ]	1.86 [1.04; 2.43]	1.69 [1.07; 2.17]	0.561
Cardiopulmonary bypass, n (%)	27 (96.4)	173 (93.0)	0.689
Off-pump CABG, n (%)	1 (3.6)	13 (6.9)	0.263
Isolated CABG, n (%)	18 (64.3)	134 (72.0)	0.263
Combined surgeries, n (%)	10 (35.7)	52 (27.9)	0.383
Aortic occlusion time, Me [LQ; UQ]	58.0 [42.0; 78.0]	55.0 [44.0; 70.0]	0.847
Total surgery duration, hours, Me [LQ; UQ]	3.5 [3.2; 4.5]	3.4 [3.2; 4.2]	0.250
CPB duration, minutes, Me [LQ; UQ]	80.0 [68.0; 117.0]	87.5 [70.5; 107.0]	0.618
Mechanical ventilation duration, hours, Me [LQ; UQ]	7.0 [5.0; 9.0]	6.9 [5.0; 10.0]	0.932
ICU duration, hours, Me [LQ; UQ]	22.0 [21.0; 43.0]	21.6 [19.6; 33.0]	0.121
ICU duration, days, Me [LQ; UQ]	0.90 [0.87;1.80]	0.90 [0.76; 1.70]	0.190
Renin–angiotensin system blockers	28 (100)	185 (99.5)	0.938
β-blockers	25 (89.3)	168 (90.3)	0.765
Calcium channel blockers	19 (67.8)	124 (66.7)	0.806
Statins	25 (89.3)	167 (89.8)	0.801
Mineralocorticoid receptor antagonists, n (%)	15 (53.6)	102 (54.8)	0.435
Oral medications for the treatment of, n (%)	22 (78.6)	133 (71.5)	0.296
Metformin, n (%)	14 (50.0)	92 (49.5)	0.765
Sulfonylurea drugs	13 (46.4)	84 (45.1)	0.533
DPP-4 inhibitors	2 (7.1)	13 (7.0)	0.939
SGLT-2 inhibitors	1 (3.6)	4 (2.7)	0.836
GLP-1 agonists	1 (3.6)	3 (1.6)	0.972
Prehospital insulin	4 (14.2)	26 (14.5)	0.804
Hospital insulin	14 (50.0)	91 (48.9)	0.704
Total insulin dose on day 1, U, Me [LQ; UQ]	38 [26; 61]	12.2 [0; 28]	<0.001

Notes: Me [LQ; UQ]—median with upper and lower quartiles, PCI—percutaneous coronary intervention, CABG—coronary artery bypass grafting, FC—functional class, CKD–EPI—Chronic formula Kidney Disease Epidemiology Collaboration, HbA1 c—glycated hemoglobin, fraction C, ICU—intensive care unit, DPP-4—dipeptidyl peptidase 4, SGLT-2—sodium–glucose cotransporter type 2; GLP-1—glucagon-like peptide 1.

**Table 2:** Association of glycemic indices with the risk of developing hospital complications after coronary artery bypass grafting: binary logistic regression results (forward LR method).

Significant complications								
						95% C.I.for EXP(B)		
	B	S.E.	Wald	df	Sig.	Exp(B)	Lower	Upper
Average glycemia	0.185	0.064	8.430	1	0.004	1.204	1.062	1.364
Constant	−2.778	0.811	11.718	1	0.001	0.062		
Serious complications								
						95% C.I.for EXP(B)		
	B	S.E.	Wald	df	Sig.	Exp(B)	Lower	Upper
Average glycemia	0.144	0.062	5.471	1	0.019	1.155	1.024	1.304
Constant	−2.285	0.785	8.477	1	0.004	0.102		

## Data Availability

Data is unavailable due to privacy restrictions.

## References

[1] Vrints C., Andreotti F., Koskinas K.C., Rossello X., Adamo M., Ainslie J., Banning A.P., Budaj A., Buechel R.R., Chiariello G.A. (2024). 2024 ESC Guidelines for the management of chronic coronary syndromes. Eur. Heart J..

[2] Ivanov S.V., Sumin A.N. (2021). Current trends in routine myocardial revascularization. Complex Issues Cardiovasc. Dis..

[3] Raza S., Sabik J.F., Ainkaran P., Blackstone E.H. (2015). Coronary artery bypass grafting in diabetics: A growing health care cost crisis. J. Thorac. Cardiovasc. Surg..

[4] Cosentino F., Grant P.J., Aboyans V., Bailey C.J., Ceriello A., Delgado V., Federici M., Filippatos G., Grobbee D.E., Hansen T.B. (2020). 2019 ESC Guidelines on diabetes, pre-diabetes, and cardiovascular diseases developed in collaboration with the EASD. Eur. Heart J..

[5] Sousa-Uva M., Head S.J., Milojevic M., Collet J.P., Landoni G., Castella M., Dunning J., Gudbjartsson T., Linker N.J., Sandoval E. (2018). 2017 EACTS Guidelines on perioperative medication in adult cardiac surgery. Eur. J. Cardiothorac. Surg..

[6] Li X., Zhou X., Wei J., Mo H., Lou H., Gong N., Zhang M. (2019). Effects of Glucose Variability on Short-Term Outcomes in Non-Diabetic Patients After Coronary Artery Bypass Grafting: A Retrospective Observational Study. Heart Lung Circ..

[7] Chen Y., Zhang H., Hou X., Li X., Qian X., Feng X., Liu S., Shi N., Zhao W., Hu S. (2021). Glycemic control and risk factors for in-hospital mortality and vascular complications after coronary artery bypass grafting in patients with and without preexisting diabetes. J. Diabetes.

[8] Li X., Hou X., Zhang H., Qian X., Feng X., Shi N., Guo R., Sun H., Feng W., Zhao W. (2023). Association between stress hyperglycaemia and in-hospital cardiac events after coronary artery bypass grafting in patients without diabetes: A retrospective observational study of 5450 patients. Diabetes Obes. Metab..

[9] Siddiqui K.M., Asghar M.A., Khan M.F., Khan F.H. (2019). Perioperative glycemic control and its outcome in patients following open heart surgery. Ann. Card. Anaesth..

[10] (2024). American Diabetes Association Professional Practice Committee. 16. Diabetes Care in the Hospital: Standards of Care in Diabetes—2024. Diabetes Care.

[11] Dedov I., Shestakova M., Mayorov A., Mokrysheva N., Andreeva E., Bezlepkina O., Peterkova V., Artemova E., Bardiugov P., Beshlieva D. (2023). Standards of Specialized Diabetes Care. Diabetes Mellit..

[12] Li X., Hou X., Zhang H., Qian X., Feng X., Shi N., Sun H., Feng W., Zhao W., Li G. (2022). Effect of early hypoglycaemia on hospitalization outcomes in patients undergoing coronary artery bypass grafting. Diabetes Res. Clin. Pract..

[13] Ogawa S., Okawa Y., Sawada K., Goto Y., Yamamoto M., Koyama Y., Baba H., Suzuki T. (2016). Continuous postoperative insulin infusion reduces deep sternal wound infection in patients with diabetes undergoing coronary artery bypass grafting using bilateral internal mammary artery grafts: A propensity-matched analysis. Eur. J. Cardiothorac. Surg..

[14] Golukhova E.Z., Lifanova L.S., Pugovkina Y.V., Grigoryan M.V., Bulaeva N.I. (2021). Should We Monitor Glucose and Biomarkers in Diabetics over Heart Surgery?. J. Clin. Med..

[15] You H., Hou X., Zhang H., Li X., Feng X., Qian X., Shi N., Guo R., Wang X., Sun H. (2023). Effect of glycemic control and glucose fluctuation on in-hospital adverse outcomes after on-pump coronary artery bypass grafting in patients with diabetes: A retrospective study. Diabetol. Metab. Syndr..

[16] Wang F., Mei X. (2024). Association of blood glucose change with postoperative delirium after coronary artery bypass grafting in patients with diabetes mellitus: A study of the MIMIC-IV database. Front. Endocrinol..

[17] Dedov I.I., Shestakova M.V., Galstyan G.R., Grigoryan O.R., Esayan R.M., Kalashnikov V.Y., Kuraeva T.L., Lipatov D.V., Mayorov A.Y., Peterkova V.A. (2015). Standards of specialized diabetes care. Diabetes Mellit..

[18] Zhang Y., Dai J., Han X., Zhao Y., Zhang H., Liu X., Li W., Ling H., Zhou X., Ying C. (2020). Glycemic variability indices determined by self-monitoring of blood glucose are associated with β-cell function in Chinese patients with type 2 diabetes. Diabetes Res. Clin. Pract..

[19] Hweidi I.M., Zytoon A.M., Hayajneh A.A., Al Obeisat S.M., Hweidi A.I. (2021). The effect of intraoperative glycemic control on surgical site infections among diabetic patients undergoing coronary artery bypass graft (CABG) surgery. Heliyon.

[20] Hulst A.H., Visscher M.J., Godfried M.B., Thiel B., Gerritse B.M., Scohy T.V., Bouwman R.A., Willemsen M.G.A., Hollmann M.W., Preckel B. (2020). Liraglutide for perioperative management of hyperglycaemia in cardiac surgery patients: A multicentre randomized superiority trial. Diabetes Obes. Metab..

[21] Sindhvananda W., Poopuangpairoj W., Jaiprasat T., Ongcharit P. (2023). Comparison of glucose control by added liraglutide to only insulin infusion in diabetic patient undergoing cardiac surgery: A preliminary randomized-controlled trial. Ann. Card. Anaesth..

[22] Shi F.H., Shen L., Yue J., Ma J., Gu Z.C., Li H., Lin H.W. (2021). Intervention by clinical pharmacists can improve blood glucose fluctuation in patients with diabetes and acute myocardial infarction: A propensity score-matched analysis. Pharmacol. Res. Perspect..

[23] Shi F.H., Yu B.B., Shen L., Xu L., Jiang Y.H., Gu Z.C., Lin H.W., Li H. (2023). The Importance of Clinical Pharmacists in Improving Blood Glucose and Lipid Levels in Patients with Diabetes and Myocardial Infarction. Diabetes Metab. Syndr. Obes..

[24] Chazal E., Morin L., Chocron S., Lassalle P., Pili-Floury S., Salomon du Mont L., Ferreira D., Samain E., Perrotti A., Besch G. (2023). Impact of early postoperative blood glucose variability on serum endocan level in cardiac surgery patients: A sub study of the ENDOLUNG observational study. Cardiovasc. Diabetol..

